# Mapping Hebbian Learning Rules to Coupling Resistances for Oscillatory Neural Networks

**DOI:** 10.3389/fnins.2021.694549

**Published:** 2021-11-08

**Authors:** Corentin Delacour, Aida Todri-Sanial

**Affiliations:** Laboratoire d'Informatique, de Robotique et de Microélectronique de Montpellier, Département de Microélectronique, Université de Montpellier, CNRS, Montpellier, France

**Keywords:** oscillatory neural network, VO_2_ device, coupled relaxation oscillators dynamics, Hopfield Neural Network, Hebbian learning rule, pattern recognition

## Abstract

Oscillatory Neural Network (ONN) is an emerging neuromorphic architecture with oscillators representing neurons and information encoded in oscillator's phase relations. In an ONN, oscillators are coupled with electrical elements to define the network's weights and achieve massive parallel computation. As the weights preserve the network functionality, mapping weights to coupling elements plays a crucial role in ONN performance. In this work, we investigate relaxation oscillators based on VO_2_ material, and we propose a methodology to map Hebbian coefficients to ONN coupling resistances, allowing a large-scale ONN design. We develop an analytical framework to map weight coefficients into coupling resistor values to analyze ONN architecture performance. We report on an ONN with 60 fully-connected oscillators that perform pattern recognition as a Hopfield Neural Network.

## 1. Introduction

Coupled oscillators have been studied for decades by scientists to describe natural phenomena (Winfree, 1967) such as the synchronization of pacemaker cells responsible for the heart beating, the synchronous behavior of insect populations, or to model neuronal activity. For instance, oscillator interactions have been shown to describe memory mechanisms and other cognitive processes in the brain (Fell and Axmacher, 2011). To characterize this variety of natural oscillations, several mathematical models (Acebrón et al., 2005; Izhikevich and Kuramoto, 2006) have been developed to explain the synchronization and phase relations in groups of coupled oscillators. Meanwhile, their massive parallel computing capability has been proved by Hoppensteadt and Izhikevich (2000), Vassilieva et al. (2011), and Parihar et al. (2017) and has raised interest in designing ONN as hardware accelerators for Artificial Neural Networks (ANN) by encoding neurons' activation in the phase between oscillators. Different types of ONN have since been developed using PLLs (Hoppensteadt and Izhikevich, 2000) or oscillators in CMOS technology (Maffezzoni et al., 2015a, 2016; Jackson et al., 2018), demonstrating pattern recognition or resolution of some optimization problems like the Traveling-Salesman-Problem (Endo and Takeyama, 1992). However, to design a competitive ONN at a large scale, a design framework is needed to establish a formalism on how to perform computations with ONN and also compare its energy efficiency with ANNs running on digital processors.

Researchers have developed oscillators by using non-linear devices such as spin-torque-oscillators (Raychowdhury et al., 2019) or with materials presenting a hysteresis resistive state to induce electrical oscillations when properly biased (Sharma et al., 2015; Wang et al., 2017). A compact device that transitions from metallic to insulating state (MIT) can be manufactured with vanadium dioxide (VO_2_) (Corti et al., 2018), and has recently become an interesting candidate to design energy-efficient relaxation oscillators. Moreover, coupled-VO_2_-based oscillators have been experimentally validated for various applications such as image saliency detection (Shukla et al., 2014), graph coloring (Parihar et al., 2017), filters in Convolutional Neural Networks (Corti et al., 2021) and implementing Hopfield Neural Networks (HNN) for pattern recognition (Corti et al., 2020).

In an HNN defined by Hopfield (1982), every neuron is connected to all the others, and synaptic weights are computed with the Hebbian rule (Hoppensteadt and Izhikevich, 2000). However, setting the right coupling element between oscillators in ONNs remains a challenge (Todri-Sanial et al., 2021). Given N fully-coupled VO_2_-oscillators, it is yet unknown how to transform the coefficients obtained analytically via the Hebbian learning rule to coupling resistor values among oscillators. Further, how can one interpret the coefficient signs *W*_*ij*_ such as positive or negative values?

For weakly coupled oscillators with sinusoidal waveform, one can use the models that exist in literature (Izhikevich and Kuramoto, 2006; Maffezzoni et al., 2016) for synaptic design. However, it is more difficult for non-linear relaxation oscillators as there is no direct mapping between models and hardware. Two VO_2_-oscillators coupled by a capacitance or a resistance have been studied (Maffezzoni et al., 2015b; Parihar et al., 2015), but to the best of our knowledge, there is not yet a formalism to map weights to coupling elements in a larger network. This formalism is a crucial step to allow large-scale ONN design exploration. A greedy approach would be to tune the coupling elements corresponding to the most negative and most positive weights and linearly interpolate all the other weight values. However, this would be impractical. It would require repeated simulations and re-tuning coupling resistances when changing any oscillator parameter; hence, it is not suitable for large-scale ONN design. Parihar et al. (2015) proposed to use capacitors or resistors to implement a negative or a positive weight, respectively. However, it would imply using twice as many components to emulate a complete signed synaptic range.

**Figure 1:**
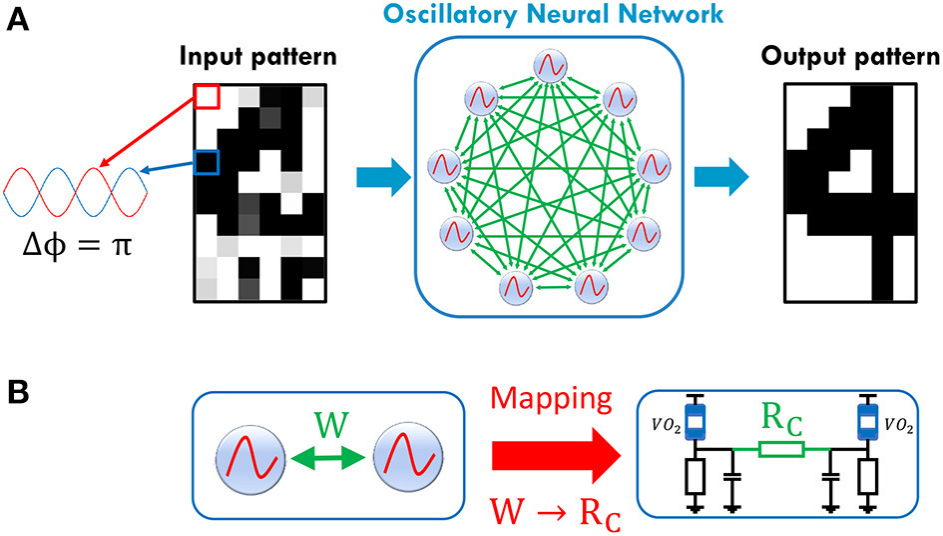
**(A)** Illustration of ONN as a Hopfield Neural Network (HNN). HNN binary activations are translated in the phase relation between oscillators and a reference (here the first oscillator). ONN stores patterns via weights between the oscillators. Patterns can be retrieved if the input pattern is sufficiently close to the stored pattern. **(B)** The proposed mapping function to map any weight to a coupling resistance, facilitating large scale ONN design.

In this work, we propose a mathematical framework to map both negative and positive Hebbian coefficient values to ONN coupling resistances, as illustrated on Figure 1. We first present a single VO_2_-oscillator followed by the dynamics of two coupled oscillators. Then, we show that adding switches between oscillators and coupling elements enhances the ONN dynamics control. Based on this simple architecture, we present the ONN computation style and how coupling resistances set the ONN memory expressed in different phase states. Next, by merging oscillators' dynamics with HNN formalism, we introduce the ONN *mapping function* that maps Hebbian coefficient values to coupling resistance values. Finally, we report on the architecture and mapping results by simulating 60 coupled VO_2_-oscillators for pattern recognition.

## 2. Materials and Methods

### 2.1. Description of the ONN Building Blocks

#### 2.1.1. General Properties

Unlike most ANNs where signals of interest are amplitudes, ONN consists of N VO_2_-oscillators coupled by resistances where the final result is given by N-1 phase relations to a reference oscillator (Corti et al., 2018). Despite this difference, there are several aspects common to any ANN that motivate our ONN study:

- The network is composed of neurons (oscillators) having input and output nodes.
- The activation function between the oscillator input and output is non-linear. For more than two oscillators, the activation function is unknown but bounded as the output phase difference of neuron *i* $\text{Δ}\phi_{i}^{out}$ is in the range [0;180°].
- ONN has a memory (coupling resistances) and can therefore be trained to achieve specific functionality.

We use these properties to implement an HNN (Hopfield, 1982) by encoding its binary outputs in the ONN phase as shown in Figure 1A. Conveniently, we can represent each oscillator's output as a black pixel if $\Delta \phi_{i}^{out}=180^{{}^{\circ}}$ or as a white pixel if $\Delta \phi_{i}^{out}=0^{{}^{\circ}}$.

#### 2.1.2. VO_2_ Device

**Figure 2:**
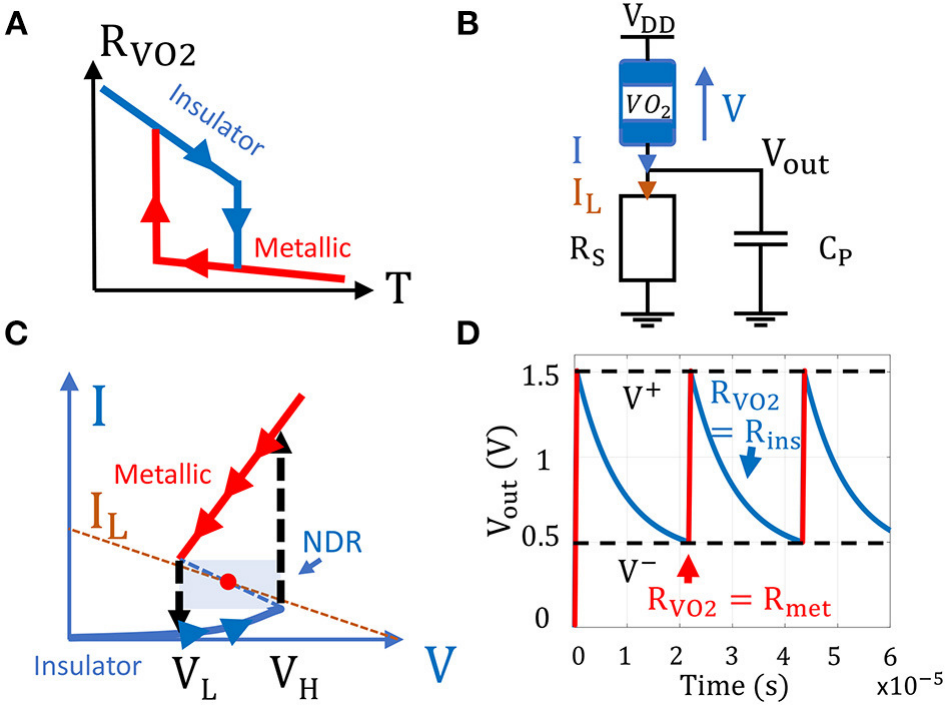
**(A)** VO_2_ resistance vs. temperature. *R*_*V*_*O*__2__ axis is in logarithmic scale. **(B)** VO_2_ oscillator circuit **(C)** VO_2_ I-V curve showing the device hysteresis behavior. When its voltage reaches a threshold *V*_*H*_, it transitions from insulating to the metallic state. To transition from a metallic to insulating state, *V* must be lower than the threshold *V*_*L*_. **(D)** If the device is biased in its Negative Differential Resistance region (NDR), its state alternates between the metallic and insulating state, producing electrical oscillations.

Vanadium dioxide is a material which presents temperature-driven phase change transitions. At room temperature, it remains semiconductor (insulating state) with a monoclinic crystal structure and transitions to a rutile metallic state when the temperature reaches a threshold (Corti et al., 2021). VO_2_ presents an hysteresis behavior as it needs to reach a lower temperature threshold to transition back to the insulating state (Figure 2A).

When a VO_2_ device is in series with a biasing load (Figure 2B), the voltage drop *V* across the device induces Joule heating which can trigger the insulating to metallic transition (IMT). To obtain the VO_2_ IV characteristic, we sweep the supply voltage which triggers IMT and MIT when *V* ≥ *V*_*H*_ and *V* ≤ *V*_*L*_, respectively. The VO_2_ hysteresis behavior appears in the IV characteristic with a typical Negative Differential Region (NDR) suitable to bias the device and produce oscillations (Figure 2C).

In this work, we use the VO_2_ compact model from Maffezzoni et al. (2015b) which reproduces the VO_2_ hysteresis along with continuous and abrupt transitions between the two states. The VO_2_ hysteresis behavior is conceptually emulated by an amplifier with positive feedback that charges or discharges a RC circuit when the VO_2_ is in metallic or insulating state, respectively. The voltage *V*_*c*_ across the capacitor commands the VO_2_ conductance *G*_*V*_*O*__2__ as:


(1) $$\begin{array}{l} G_{VO_{2}}\left(t\right)=\frac{1-V_{c}\left(t\right)}{R_{ins}}+\frac{V_{c}\left(t\right)}{R_{met}} \end{array}$$

Where *R*_*ins*_ and *R*_*met*_ are the VO_2_ resistances in insulating and metallic state, respectively. The dynamics of the VO_2_ conductance is given by the RC circuit where τ_0_ is its time constant modeling the transition time of the VO_2_:


(2) $$\begin{array}{l} \tau_{0}\frac{dV_{c}\left(t\right)}{dt}+V_{c}\left(t\right)=1-V_{0}\left(t\right) \end{array}$$

*V*_0_(*t*) is the output of the positive feedback amplifier (gain α) and is expressed as:


(3) $$V_{0}(t)=\frac{1}{2}[1+\tanh(2\alpha ((V_{H}-V_{L})V_{0}(t)+V_{L}-V(t)))]$$

#### 2.1.3. VO_2_ Oscillator Circuit and Dynamics

We bias the VO_2_ device with a series resistor *R*_*S*_ to obtain a compact relaxation oscillator. To produce oscillations, the load line *I*_*L*_ set by *V*_*DD*_ and *R*_*S*_ must intercept the VO_2_ I-V curve in its NDR to obtain an unstable fixed point (Figure 2C). The VO_2_ device state hence alternates between the metallic and insulating state. When the VO_2_ device is in the insulating state, the parallel capacitance *C*_*P*_ at the output node discharges through *R*_*S*_ until the VO_2_ voltage reaches *V*_*H*_ and transitions to the metallic state. Then, *C*_*P*_ charges through the VO_2_ device until its voltage reaches *V*_*L*_ and a new cycle begins (Figure 2D).

We use circuit parameters depicted in Table 1. In this case, the load resistance *R*_*S*_ is 20 times larger than the VO_2_ metallic resistance *R*_*met*_; thus, the capacitance charge time is much faster than its discharge (Figure 2D).

**Table 1:** List of parameters used for simulations in this work.

**Parameter**	**Value**
*V*_*DD*_	2.5 V
*R*_*S*_	20 kΩ
*C*_*P*_	500 pF
*R*_*ins*_	100.2 kΩ
*R*_*met*_	0.99 kΩ
*V*_*L*_	1 V
*V*_*H*_	1.99 V
α	200
τ_0_	10 ns
$V^{+}=V_{DD}-V_{L}$	1.5 V
$V^{-}=V_{DD}-V_{H}$	0.501 V
*T*_*osc*_	21.6μs

Oscillator's dynamics can be described by Kirchoff's law as:


(4) $$\begin{array}{l} C_{P}\frac{dV_{out}\left(t\right)}{dt}=\left(V_{DD}\left(t\right)-V_{out}\left(t\right)\right)G_{VO_{2}}\left(t\right)-\frac{V_{out}\left(t\right)}{R_{S}} \end{array}$$

Note that despite the first order differential equation, oscillations can occur as Equations (1–3) describe the hysteresis behavior of *G*_*V*_*O*__2__(*t*). To solve the oscillator dynamics, we start from an initial insulating VO_2_ state and solve numerically on Matlab the system of Equations (1–4) by using Euler's method and Newton-Raphson's algorithm for non-linear Equation (3). Figure 2D shows an example where *V*_*out*_(*t* = 0) = 0*V*, *G*_*V*_*O*__2__(*t* = 0) = 1/*R*_*ins*_, and *V*_*DD*_(*t* = 0) = 2.5*V*.

#### 2.1.4. Initialization of Two Coupled Oscillators

Two coupled oscillators represent the smallest ONN and serve as the building block for large-scale ONN. To provide input to the ONN, we delay the second oscillator *V*_*DD*_ starting time with respect to the first oscillator (reference oscillator) to set an initial phase relation between them. Assuming oscillators have the same period *T*_*osc*_, we can translate the input delay Δ*t*_*init*_ as an initial phase relation as:


(5) $$\begin{array}{l} \Delta \phi_{init}=\frac{\Delta t_{init}}{T_{osc}}2\pi \end{array}$$

**Figure 3:**
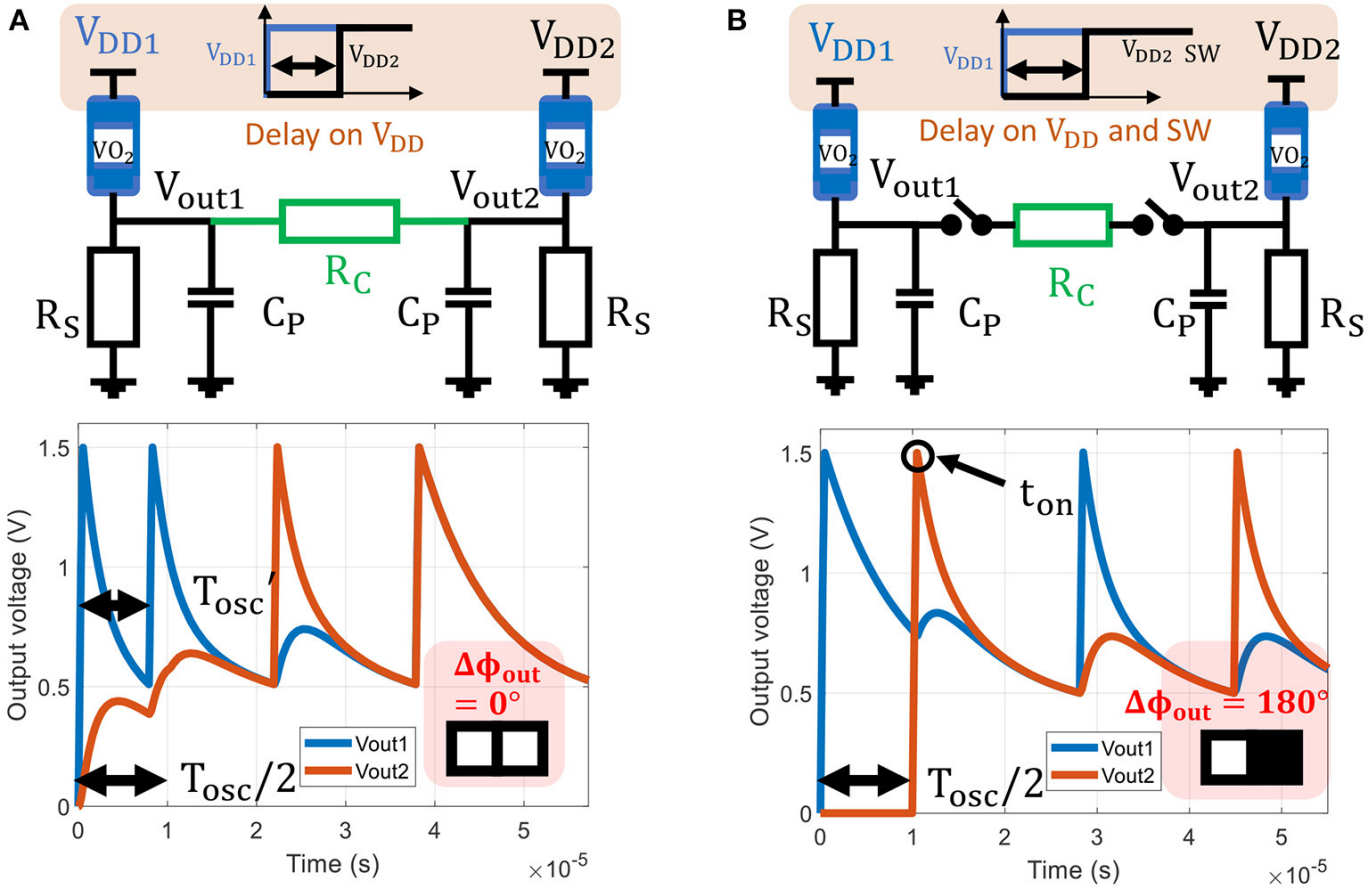
**(A)** Two oscillators are coupled by a resistance *R*_*C*_=10 kΩ without coupling switches in between. *V*_*DD*2_ is turned-on 0.5*T*_*osc*_ after *V*_*DD*1_ to set an initial phase of 180°. However, for *t* < 0.5*T*_*osc*_ the first oscillator period is decreased due to the shunt *R*_*C*_ at its output node, and we cannot control the initial phase difference. The two oscillators are in-phase after convergence. **(B)** Two oscillators coupled with resistance *R*_*C*_=10 kΩ and coupling switches between isolate the oscillators during initialization. This time, the 0.5*T*_*osc*_ input delay sets desired 180° initial phase state. Here, the switches are closed at *t*_*on*_ = 0.5*T*_*osc*_ + *t*_*c*_ such that $V_{out2}\left(t_{on}\right)=V^{+}$. The two oscillators converge to an 180° phase state relation.

However, if the two oscillators are always connected, they might have different oscillation periods during initialization. Therefore, their initial phase relation cannot be represented as a proportion of *T*_*osc*_ (5). For example, as shown in Figure 3A, the second oscillator starts Δ*t*_*init*_ = 0.5*T*_*osc*_ after the first one to set an initial phase relation Δϕ_*init*_ = π. For *t* < Δ*t*_*init*_, the second oscillator is off, and its output node is floating. Therefore, during this time, the equivalent load resistance of the first oscillator is *R*_*S*_//*R*_*C*_, which induces a shorter period of oscillation $T_{osc}^{\prime }<T_{osc}$ and hence no control on the initial phase.

We introduce switches between each oscillator and coupling elements as in Figure 3B to tackle this lack of control. We let each oscillator switch freely with a known oscillation period *T*_*osc*_ before coupling them at *t*_*on*_. Their dynamics can be expressed by Equation (4) and the initial conditions will be known at *t*_*on*_. ONN initialization is improved at the cost of one additional switch per oscillator, which can be achieved with a transfer gate.

#### 2.1.5. Dynamics of Two Coupled Oscillators

To predict the output phases and demonstrate ONN ability to store information, we express the dynamics of the two coupled oscillators using Kirchhoff's laws:


(6) $$\begin{array}{l} \{\begin{array}{c} C_{P}\frac{dV_{out1}\left(t\right)}{dt}=\left(V_{DD1}\left(t\right)-V_{out1}\left(t\right)\right)G_{VO_{2}}^{1}\left(t\right)-\frac{V_{out1}\left(t\right)}{R_{S}}+I_{c1}\\ C_{P}\frac{dV_{out2}\left(t\right)}{dt}=\left(V_{DD2}\left(t\right)-V_{out2}\left(t\right)\right)G_{VO_{2}}^{2}\left(t\right)-\frac{V_{out2}\left(t\right)}{R_{S}}+I_{c2} \end{array} \end{array}$$

**Figure 4:**
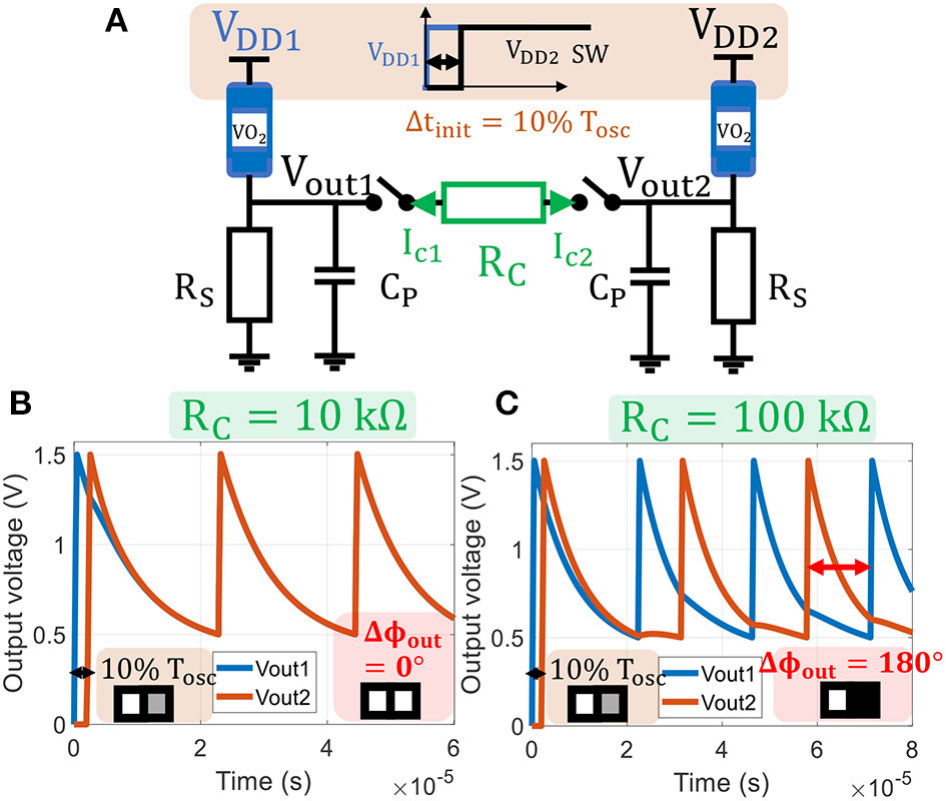
**(A)** Two identical VO_2_ oscillators are coupled with a resistance *R*_*C*_ and switches. *V*_*DD*2_ starting time and coupling time *t*_*on*_ are delayed by 0.1*T*_*osc*_ with respect to the first oscillator, representing a light-gray second pixel as ONN input. **(B)** Output voltages for *R*_*C*_ = 10*kΩ*: the oscillators converge to an in phase state (0°, 0°) and the corresponding output pattern corresponds to two white pixels. **(C)** Output voltages for *R*_*C*_ = 100*kΩ*: the oscillators are out-of-phase (0°, 180°) and the output pattern corresponds to a white and a black pixels.

Currents are *I*_*c*1_ = −*I*_*c*2_ representing the coupling element's current flow. As for the single oscillator case, we numerically solve (Equation 6) along with VO_2_ (Equations 1–3). Figure 4 shows a simulation where *V*_*DD*2_ is turned on 0.1*T*_*osc*_ after *V*_*DD*1_ which initializes a light-gray pixel for oscillator 2 input image. For a small coupling resistance, *R*_*C*_=10 kΩ, ONN converges to a stable state with both oscillators in-phase (0°,0°). Whereas, for *R*_*C*_=100 kΩ, ONN converges to out-of-phase (0°,180°). In the next subsection, we study the role of *R*_*C*_ on ONN memory and investigate how to retrieve a stored pattern by applying an input delay Δ*t*_*init*_. This formulation is the core of our proposed *mapping function* to translate Hebbian coefficients to ONN coupling resistances.

#### 2.1.6. Memory of Two Coupled Oscillators

**Figure 5:**
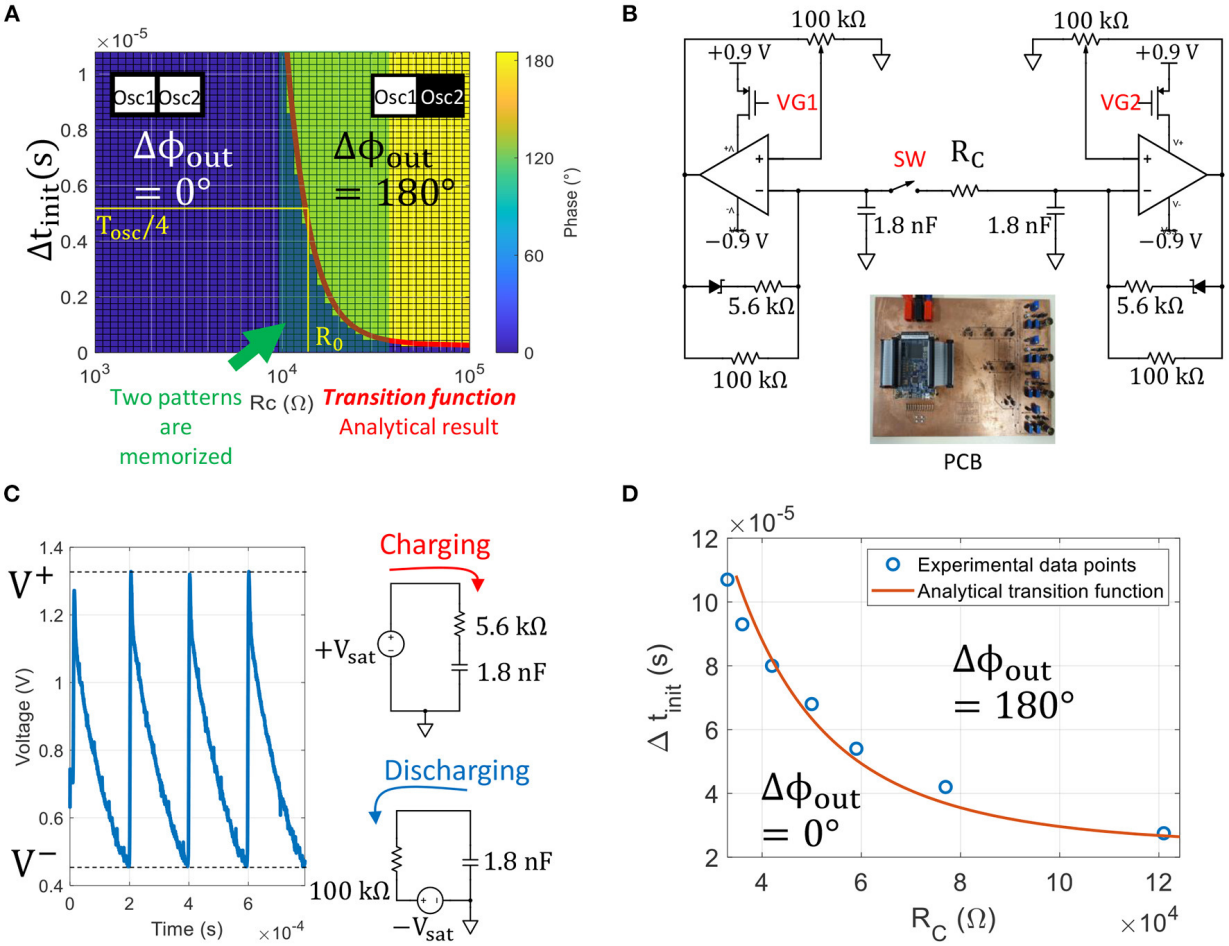
**(A)** Plot showing the phase relation between two oscillators for every set of parameters (*R*_*C*_, Δ*t*_*init*_). As expected, small coupling resistances tend to pull the oscillator phase together, whereas large coupling resistances push the phase away. The green region shows the coupling resistance range 10kΩ < *R*_*C*_ < 40kΩ in which two patterns (0° and 0°) and (0° and 180°) are memorized and can be retrieved by adjusting the input delay. The red curve is our analytical model describing the *transition* between the two phase states in the plan (*R*_*C*_, Δ*t*_*init*_), and plays a major role in the ONN ability to memorize patterns. **(B)** Experimental set-up of two coupled relaxation oscillators based on MCP6001 OPAs. We delay VG2 with respect to VG1 by Δ*t*_*init*_ to set the initial phase, and we close SW after initialization. **(C)** Experimental oscillating waveform and equivalent circuits during charge and discharge of the output capacitor. **(D)** Experimental phase transition curve and analytical model in plain line.

We solve numerically (6) and extract the output phase relation between oscillators. Figure 5A shows the simulation results. As already observed by Corti et al. (2018), a large coupling resistance *R*_*C*_ > 40 kΩ induces oscillators in out-of-phase relation (0°, 180°) for any input delay, whereas a small coupling resistance *R*_*C*_ < 10kΩ aligns oscillators in-phase (0°, 0°) for any input delay.

In contrast, we examine the region between these two ranges, highlighted in the center of Figure 5A. We observe that for 10kΩ ≤ *R*_*C*_ ≤ 40kΩ both states co-exist and oscillators store two patterns (0°, 0°) and (0°, 180°) that can be retrieved by adjusting the input delay. The line *transition function* between in-phase and out-of-phase regions represents our analytical function for ONN memory with respect to coupling resistance and initialization. It is defined as:


(7) $$\begin{array}{l} \begin{array}{c} \zeta :R_{C}\to \Delta t_{transit} \end{array} \end{array}$$

With Δ*t*_*transit*_ the initial delay such that:


(8) $$\begin{array}{l} \Delta t_{transit}=\zeta \left(R_{C}\right)|\{\begin{array}{l} \Delta t_{init}<\Delta t_{transit}\Rightarrow \Delta \phi_{out}=0^{{}^{\circ}}\\ \Delta t_{init}\geq \Delta t_{transit}\Rightarrow \Delta \phi_{out}=180^{{}^{\circ}} \end{array} \end{array}$$

To confirm the existence of ζ(*R*_*C*_), we emulate VO_2_ oscillators with off-the-shelf components on a Printed Circuit Board (PCB) and we reproduce the experiment of two coupled oscillators. The relaxation oscillator circuit consists of an inverting Schmitt trigger (Schmitt, 1938) Operational Amplifier (OPA) that implements the VO_2_ hysteresis behavior (Figure 5B). The OPA saturates to +*V*_*sat*_ and −*V*_*sat*_ while the 1.8 nF output capacitor charges and discharges, respectively. Figure 5C shows the voltage across the output capacitor for a decoupled oscillator. Similarly to a VO_2_ oscillator, the OPA transitions to another state when the voltage across the output capacitor reaches *V*^+^ or *V*^−^. The 5.6 kΩ resistor implements the metallic VO_2_ resistance, whereas the 100 kΩ resistor corresponds to the load *R*_*S*_. For a fixed oscillating period of *T*_*osc*_=200 μs, we vary *R*_*C*_ and we measure Δ*t*_*transit*_ values that define the experimental transition function ζ(*R*_*C*_) (Figure 5D). There is a good match between experimental ζ(*R*_*C*_) data points and the analytical transition function derived in next subsection. Such formulation ζ(*R*_*C*_) is of interest as it represents a closed-form representation of ONN memory instead of repeating numerical simulations for different oscillator parameters.

#### 2.1.7. Phase Transition Function for Two Coupled Oscillators

The phase transition function has already been observed (Nez et al., 2021) but to the best of our knowledge, no closed-form expression has ever been reported. To obtain the transition function, we solve node voltage equations analytically for two coupled oscillators during initialization (see Supplementary Material). We derive oscillator outputs as:


(9) $$\begin{array}{l} \Delta V=V_{out2}-V_{out1}=\left(V_{out2}^{0}-V_{out1}^{0}\right)\exp\left(-\frac{t}{\tau^{\prime }}\right) \end{array}$$

**Figure 6:**
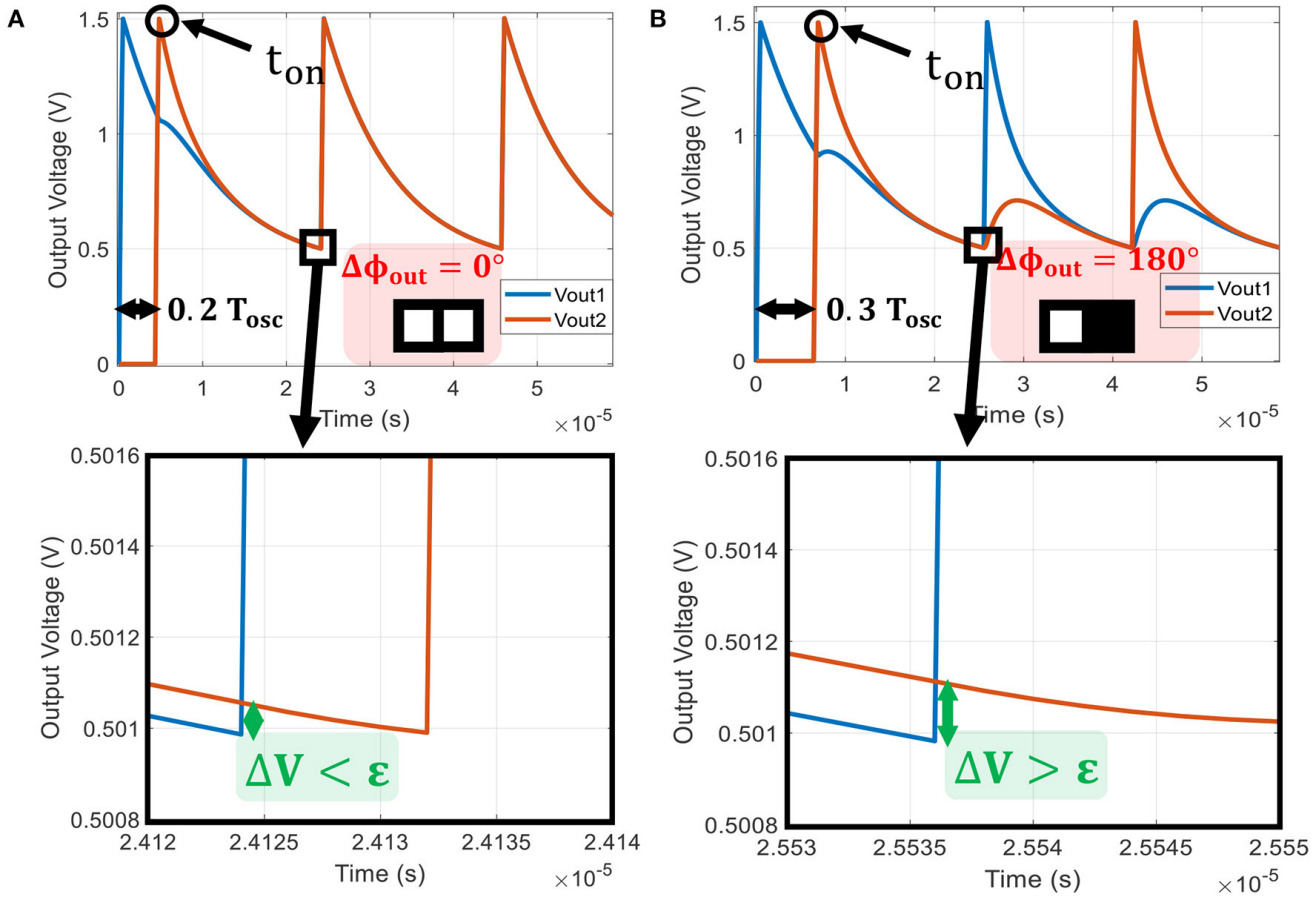
Two identical oscillators coupled by *R*_*C*_ = 12kΩ. **(A)** The second oscillator is turned on at 0.2 *T*_*osc*_ after the first one. By zooming on the waveform when the first oscillator reaches *V*^−^, we observe a voltage difference Δ*V* < ϵ. Therefore, the oscillators converge to an in-phase state. **(B)** The second oscillator is turned-on 0.3 *T*_*osc*_ after the first one. In this case, we observe a voltage difference Δ*V* > ϵ and the oscillators converge to an out-of-phase state.

$V_{out1}^{0}$ and $V_{out2}^{0}$ are the initial voltages when oscillators are coupled and τ′ is defined in Supplementary Material Equation (S18). Equation (9) describes both oscillator output voltages attracted via the coupling resistance *R*_*C*_. If the coupling is strong enough (small *R*_*C*_), both oscillators are rapidly pulled together with a speed determined by τ′. If Δ*V* < ϵ (ϵ defined in Supplementary Material Equation S22) before reaching the VO_2_ threshold *V*^−^, then both oscillators will transition to low resistive states, and the exponential term in Equation (9) will keep the two voltages locked. This concept is illustrated in Figure 6A when both oscillators are in-phase. However, if *V*_*out*1_ reaches *V*^−^ before *V*_*out*2_ such that Δ*V* > ϵ as in Figure 6B, the first oscillator transitions to a low resistance state (metallic state) while the other oscillator is still in high resistance state (insulating state). The two oscillators are then in opposite states, and this leads to out-of-phase relation.

Thus, to obtain the transition function ζ (Equation 7), we study the case when both Δ*V* = ϵ and $V_{out1}=V^{-}$ conditions are fulfilled (see Supplementary Material for details). By combining (Equations S17, S24, and S25 in Supplementary Material) we derive coupling resistance as:


(10) $$\begin{array}{l} R_{C}=2\frac{R_{S}R_{ins}}{R_{S}+R_{ins}}\frac{\log\left(\frac{V^{-}-V_{std}^{ins}}{V_{out1}^{0}/2+V_{out2}^{0}/2-V_{std}^{ins}}\right)}{\log\left(\frac{\epsilon \left(R_{C}\right)}{V_{out2}^{0}-V_{out1}^{0}}\right)-\log\left(\frac{V^{-}-V_{std}^{ins}}{V_{out1}^{0}/2+V_{out2}^{0}/2-V_{std}^{ins}}\right)} \end{array}$$

where $V_{std}^{ins}$ is defined in Supplementary Material Equation (S5). Finally, we introduce (Equation S9 in Supplementary Material) into Equation (10), and obtain a relation between *R*_*C*_ and Δ*t*_*transit*_. Note that ϵ is a function of *R*_*C*_, thus we cannot solve (10) analytically. Instead, we numerically solve (Equation 10) using Newton-Raphson's algorithm for Δ*t*_*transit*_ values. We finally obtain *R*_*C*_ values that describe the inverse of the transition function as:


(11) $$\begin{array}{l} \begin{array}{c} \zeta^{-1}:\Delta t_{transit}\to R_{C} \end{array} \end{array}$$

Transition function ζ is plotted as the curve line (red line) in Figure 5A, and there is an excellent fit between our analytical model, simulations (transition region between dark and light green in (*R*_*C*_, Δ*t*_*init*_) plan) and the transition curve obtained experimentally with off-the-shelf relaxation oscillators (Figure 5D). In addition, we can now extract the coupling resistor *R*_0_ that corresponds to a neutral synaptic connection *W* = 0. As by definition, both output phase states can equally occur for *W* = 0, we extract *R*_0_ as:


(12) $$\begin{array}{l} R_{0}=\zeta^{-1}\left(\Delta t_{init}=T_{osc}/4\right) \end{array}$$

Finally, based on the transition function, we predict the final phase relation as:


(13) $$\begin{array}{l} \Delta \phi_{out}=180^{{}^{\circ}}\left(sign(R_{C}-\zeta^{-1}\left(\Delta t_{init}\right))+1\right)/2 \end{array}$$

Analogous to ANNs, Equation (13) can be thought of as oscillator's activation function. Because, it provides the oscillator's output phase based on its input phase (set by Δ*t*_*init*_; Equation 5) and the weight implemented by *R*_*C*_.

#### 2.1.8. Impact of VO_2_ Parameters Variations on the Phase Transition Function

**Figure 7:**
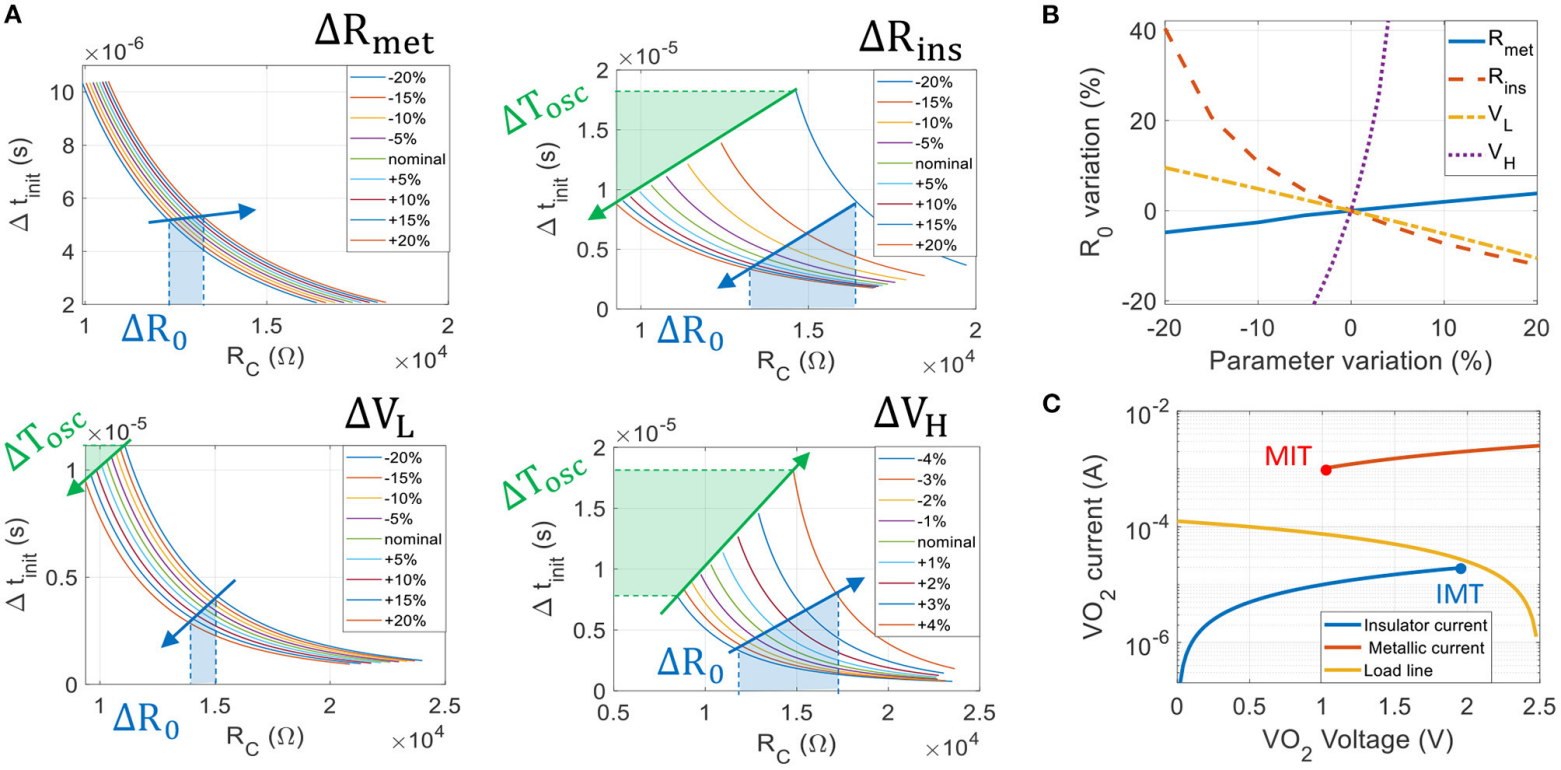
**(A)** Phase transition curves ζ(*R*_*C*_) when varying VO_2_ parameters *R*_*met*_, *R*_*ins*_, *V*_*L*_ from –20 to +20%, and *V*_*H*_ from –4 to +4% for circuit parameters listed in Table 1. Left hand side of the transition curve corresponds to inputs (Δ*t*_*init*_, *R*_*C*_) inducing $\Delta \phi_{out}=0^{{}^{\circ}}$ whereas right hand side corresponds to $\Delta \phi_{out}=180^{{}^{\circ}}$ phase region. *V*_*H*_ and *R*_*ins*_ variations correspond to IMT point variations and have the most detrimental impact on the transition function variations. The oscillating period almost doubles due to IMT variations. **(B)** Variations of the neutral synaptic resistance *R*_0_ with respect to VO_2_ parameters' variations. *R*_0_ is very sensitive to *V*_*H*_ as –4 and +4% *V*_*H*_ variations induce –20 and +40% *R*_0_ variations, respectively. **(C)** The ONN sensitivity to IMT point is mainly due to the load line *I*_*L*_ = (*V*_*DD*_−*V*)/*R*_*S*_ placed close to IMT point. Any IMT variation greatly impacts the oscillators' dynamics defined by *C*_*P*_*dV*/*dt* = *I*_*L*_−*I*.

Fabricating reliable VO_2_ devices is challenging (Corti et al., 2019) and ONN experiments with VO_2_ are currently limited to few devices because of device variability (Shukla et al., 2016). Here, we study the impact of VO_2_ variability on the ability to phase-lock and on the synaptic range. The transition function ζ(*R*_*C*_) defines the boundary between two phase regions, and allows direct identification of the neutral coupling resistor *R*_0_ corresponding to the weight *W* = 0 (Equation 12). Thus, we use ζ and *R*_0_ as metrics to assess the impact of VO_2_ parameters' variations. We apply relative variations on VO_2_ parameters one at a time from –20% up to +20%, as shown in Figure 7A. Note that we vary *V*_*H*_ from –4% up to +4% only, as for larger positive variations oscillations do not occur (load line crosses the insulating branch and forms a fixed point). For all cases, we discretize the whole input space (*R*_*C*_, Δ*t*_*init*_) and perform multiples transient simulations to extract the new phase regions. Then, we numerically solve (Equation 10) with the new sets of parameters and we verify that the transition function ζ matches the phases boundary obtained via transient simulations, as in Figure 5A.

Figure 7A shows the set of phase transition curves obtained when varying *R*_*met*_, *R*_*ins*_, *V*_*L*_ and *V*_*H*_. Note that our current formalism assumes matched oscillators and hence, variations are applied to both coupled oscillators. For all curves, the maximum Δ*t*_*init*_ value corresponds to an input delay of *T*_*osc*_/2 and shows the oscillation period variation (highlighted in green in Figure 7A). Finally, we extract *R*_0_ for each configuration (Figure 7B). We observe that variations on the IMT point (defined by *R*_*ins*_ and *V*_*H*_) induce the largest *T*_*osc*_ and *R*_0_ variations. With our biasing set by *R*_*S*_ and *V*_*DD*_ (Table 1), the most sensitive VO_2_ parameter is *V*_*H*_ as +4 and –4% *V*_*H*_ variations induces +40 and –20% *R*_0_ variations, respectively. As the dynamic of the voltage *V* across the VO_2_ device is given by *C*_*P*_*dV*/*dt* = *I*_*L*_−*I*, we believe this sensitivity is mainly due to the load line that passes very close to the IMT point on the VO_2_ *IV* characteristic (Figure 7C). In this case near IMT, *I*_*L*_(*V*_*H*_)−*I*(*V*_*H*_) is small and the voltage “slows down” and is very sensitive to any IMT variation. When applying –4% up to +4% *V*_*H*_ variations, the oscillating period *T*_*osc*_ almost doubles (same remark with –20 and +20% *R*_*ins*_ variations). Ideally, we would then place the load line at equal distances between MIT and IMT points (*I*(*V*_*L*_) − *I*_*L*_(*V*_*L*_) ≈ *I*_*L*_(*V*_*H*_) − *I*(*V*_*H*_)) to homogenize the impact of VO_2_ variations. However, we show in the next subsection that such biasing would prevent binary phase locking and that resistively coupled oscillators need a very asymmetric waveform to phase-lock to 180°.

#### 2.1.9. Impact of Oscillators' Waveform Shape on ONN Phase-Locking

Oscillators' circuit parameters listed in Table 1 influence the oscillating frequency, amplitude and waveform shape. The oscillating waveform shape has a major influence on ONN phase-locking capability and has been studied for PLL-based ONNs by Hoppensteadt and Izhikevich (2000). Here, we study the impact of the oscillating waveform shape on the capability for pairs of oscillators to lock to the 180° phase state. We characterize the oscillating waveform shape with the ratio τ_*d*_/τ_*c*_, where τ_*d*_ and τ_*c*_ are the discharging and charging time constant, respectively (defined in Supplementary Material Equations S6, S7). Our transition function ζ links ONN phase-locking properties to the metric τ_*d*_/τ_*c*_, as ζ only depends on oscillators' internal parameters.

**Figure 8:**
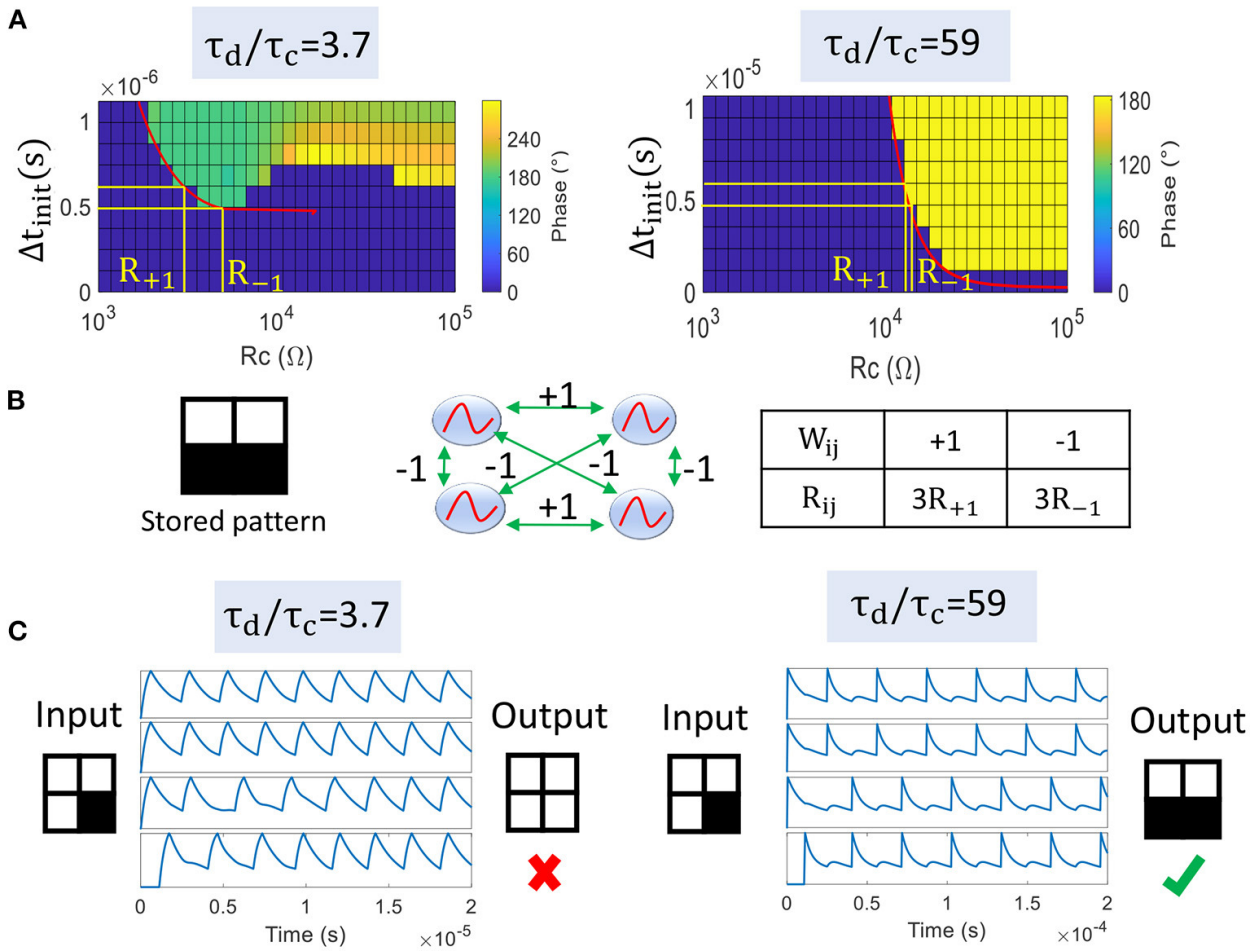
**(A)** Phase plots showing Δϕ_*out*_ with respect to Δ*t*_*init*_ and coupling resistance *R*_*C*_ between two oscillators. For τ_*d*_/τ_*c*_ = 3.7, 180° phase state is not reachable for low Δ*t*_*init*_ values. In contrast for τ_*d*_/τ_*c*_ = 59, 180° phase-locking can occur for any Δ*t*_*init*_ value for large *R*_*C*_. The red line is our analytical model ζ(*R*_*C*_) and captures well the boundary between phase regions. **(B)** Four coupled oscillators store a pattern composed of 2 white and 2 black pixels. Positive and negative weights are mapped to 3x*R*_+1_ and 3x*R*_−1_, respectively. **(C)** ONN inference for τ_*d*_/τ_*c*_ = 3.7 and τ_*d*_/τ_*c*_ = 59. The first configuration leads to a wrong in-phase relationship for all oscillators. In the latter case, ONN retrieves the correct stored pattern.

We reproduce the previous simulation with two coupled oscillators to extract the output phase regions for different load resistances *R*_*S*_ that set τ_*d*_/τ_*c*_ (Figure 8A). Note that we also could have varied VO_2_ parameters such as *R*_*met*_, but instead we consider the same device. For τ_*d*_/τ_*c*_ = 3.7 (*R*_*S*_ = 3 kΩ), we observe that the two oscillators cannot lock to $\Delta \phi_{out}=180^{{}^{\circ}}$ for small Δ*t*_*init*_ values. In other words, the phase state $\Delta \phi_{out}=180^{{}^{\circ}}$ stored by a large *R*_*C*_ cannot be fully recovered. This can be an issue for some pairs of oscillators that need an out-of-phase relationship for any input delay.

If τ_*d*_/τ_*c*_ = 59 (*R*_*S*_ = 20 kΩ), the charging time is much smaller than the discharging time and the oscillating waveform becomes very asymmetrical. Interestingly, this configuration enlarges the 180° phase region and $\Delta \phi_{out}=180^{{}^{\circ}}$ is reachable for any Δ*t*_*init*_ value for large *R*_*C*_. Our analytical model ζ(*R*_*C*_) predicts the correct boundary between the two phase regions (red plain lines in Figure 8A).

We study a simple case where 4 VO_2_-oscillators are coupled by resistances to store a single pattern (Figure 8B). Based on transition functions obtained for 2 coupled oscillators, we compute coupling resistances *R*_+1_ and *R*_−1_ that correspond to synaptic coefficients +1 and –1, respectively. We set *R*_+1_ and *R*_−1_ around *R*_0_ as $R_{+1}=\zeta^{-1}\left(T_{osc}/4+T_{osc}/8\right)$ and $R_{-1}=\zeta^{-1}\left(T_{osc}/4-T_{osc}/8\right)$, respectively. Then, we scale coupling resistances as 3x*R*_+1_ and 3x*R*_−1_ as every oscillator is connected to 3 others (Figure 8B). We notice that ONN with τ_*d*_/τ_*c*_ = 59 retrieves the correct stored pattern whereas ONN with τ_*d*_/τ_*c*_ = 3.7 produces a wrong output (Figure 8C). In the latter case, we observe that all oscillators converge to an in-phase relationship. We believe this wrong behavior is mainly due to the small τ_*d*_/τ_*c*_ value for which it is less likely ONN converges to $\Delta \phi_{out}=180^{{}^{\circ}}$, as described by the transition function ζ.

**Figure 9:**
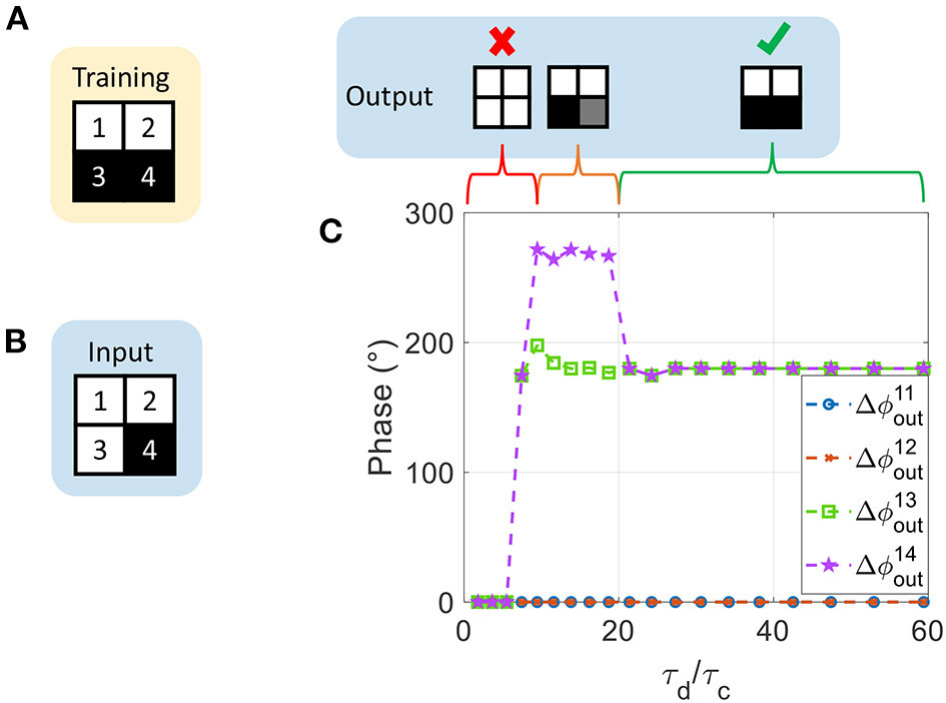
**(A)** Training pattern stored by the ONN. **(B)** ONN input pattern. **(C)** Output phase with respect to τ_*d*_/τ_*c*_. For τ_*d*_/τ_*c*_ < 7, all oscillators converge to a wrong in-phase relationship. For 7 ≤ τ_*d*_/τ_*c*_ < 20, the fourth oscillator locks to a 270° phase state. A very asymmetrical waveform such that τ_*d*_/τ_*c*_ ≥ 20 leads to a correct binary phase-locking.

Figure 9 shows results of the same experiment for τ_*d*_/τ_*c*_ varied from 1.8 up to 59 (obtained for 2 kΩ ≤ *R*_*S*_ ≤ 20 kΩ). We observe that τ_*d*_/τ_*c*_ > 20 is required to retrieve the correct pattern. Interestingly for τ_*d*_/τ_*c*_ ≤ 20, there are cases where the fourth oscillator locks to a phase state around 270°. 270° phase value is also obtained in the phase plot between two coupled oscillators, such as on the left-hand side of Figure 8A. This phenomenon would allow more than two phase values but is not captured by our current formalism. In contrast, we set τ_*d*_/τ_*c*_ to high values (59 in this work) to ensure binary 0° and 180° phase locking.

To implement a large-scale neural network such as HNN with an ONN, we need a systematic approach to map the weights to ONN coupling resistances. In the next section, we exploit some HNN features and our knowledge of two coupled oscillators to propose a *mapping function*.

### 2.2. ONN Weight Mapping

#### 2.2.1. Applying HNN Formalism to ONN

We exploit HNN formalism to build an analogous representation in ONN. For equivalence, we treat HNN neurons similar to ONN oscillators. Such as, we consider a neuron *i* with two possible states *S*_*i*_ that can be thought of as equivalent to ONN oscillators with 0° or 180° phase relations as:


(14) $$\begin{array}{l} S_{i}=\{\begin{array}{c} +1\\ -1 \end{array}\Leftrightarrow \Delta \phi_{i}=\{\begin{array}{l} 0^{{}^{\circ}}\\ 180^{{}^{\circ}} \end{array} \end{array}$$

**Figure 10:**
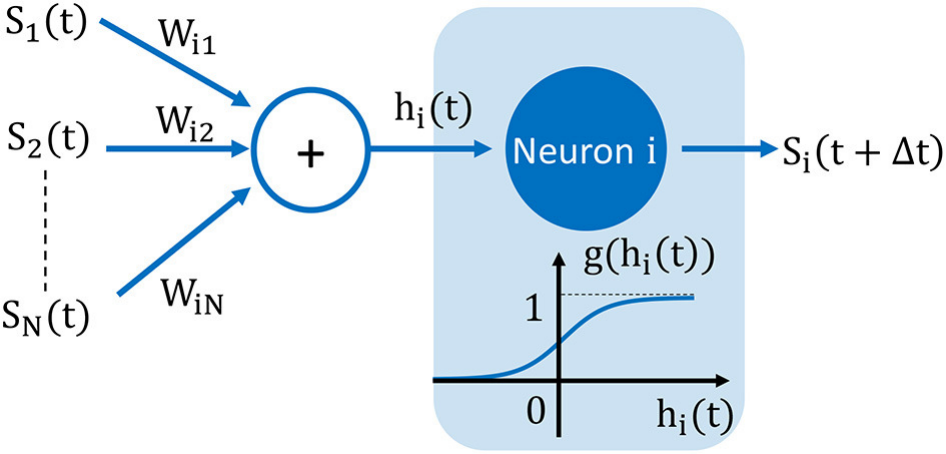
Model of artificial neuron used to construct our mapping function μ_*N*_. The neuron's output state *S*_*i*_(*t*) is either +1 or –1 and is dynamically updated at each time-step Δ*t* according to the sigmoid activation function *g*. Here, *g* gives the probability to have one of the two states at *t* + Δ*t* for a given input weighted sum *h*_*i*_(*t*).

In HNN, each neuron output state is dynamically determined by a sigmoid activation function *g*(*x*) = (tanh(β*x*) + 1)/2 (with β a positive parameter) (Gerstner et al., 2014) and shown in Figure 10. For a neuron *i*, *g* gives the probability to reach one of the two states at *t* + Δ*t* for a given input weighted sum *h*_*i*_(*t*) as


(15) $$\begin{array}{l} P\left(S_{i}\left(t+\Delta t\right)=+1|h_{i}\left(t\right)\right)=g\left(h_{i}\left(t\right)\right) \end{array}$$

with


(16) $$\begin{array}{l} h_{i}\left(t\right)=\overset{N}{\underset{j=1}{\sum }}W_{ij}S_{j}\left(t\right) \end{array}$$

In ONNs, Equation (15) would represent the probability of oscillator *i* to be in-phase with the reference at time-step Δ*t*. For two oscillators case, the weighted input sum of the second oscillator is given by:


(17) $$\begin{array}{l} h_{2}\left(t\right)=W_{21}S_{1}\left(t\right)=W_{21} \end{array}$$

Then, the probability of the second oscillator to be in-phase with the reference can be derived by Equations (15) and (17), as:


(18) $$\begin{array}{l} P\left(S_{2}\left(t+\Delta t\right)=+1|h_{2}\left(t\right)\right)=P_{inphase}=g\left(W_{21}\right) \end{array}$$

#### 2.2.2. Mapping Function

Here, we apply the above definitions to derive a mapping function using the HNN formalism, as:


(19) $$\begin{array}{l} \mu_{N}:W_{ij}\to R_{ij} \end{array}$$

where, normalized weights are −1 ≤ *W*_*ij*_ ≤ 1 and *N* is ONN size. Before scaling to N oscillators, we derive a mapping function μ_2_ for two coupled oscillators. The unifying step between HNN and ONN is the recasting of the phase transition curve, ζ as the probability *P*_*inphase*_ for a given coupling resistance *R*_*C*_. In ONNs, the input delay Δ*t*_*init*_ can be considered as a uniform random variable taking values between 0 and *T*_*osc*_/2 and the transition function ζ would give the probability *P*_*inphase*_ (for example for *R*_*C*_ > 10*kΩ*):


(20) $$\begin{array}{l} P_{inphase}=\zeta \left(R_{C}\right)\frac{2}{T_{osc}} \end{array}$$

and by Equations (18) and (20), we finally obtain


(21) $$\begin{array}{l} \mu_{2}\left(W_{21}\right)=R_{C}=\zeta^{-1}\left(\frac{T_{osc}}{2}g\left(W_{21}\right)\right) \end{array}$$

**Figure 11:**
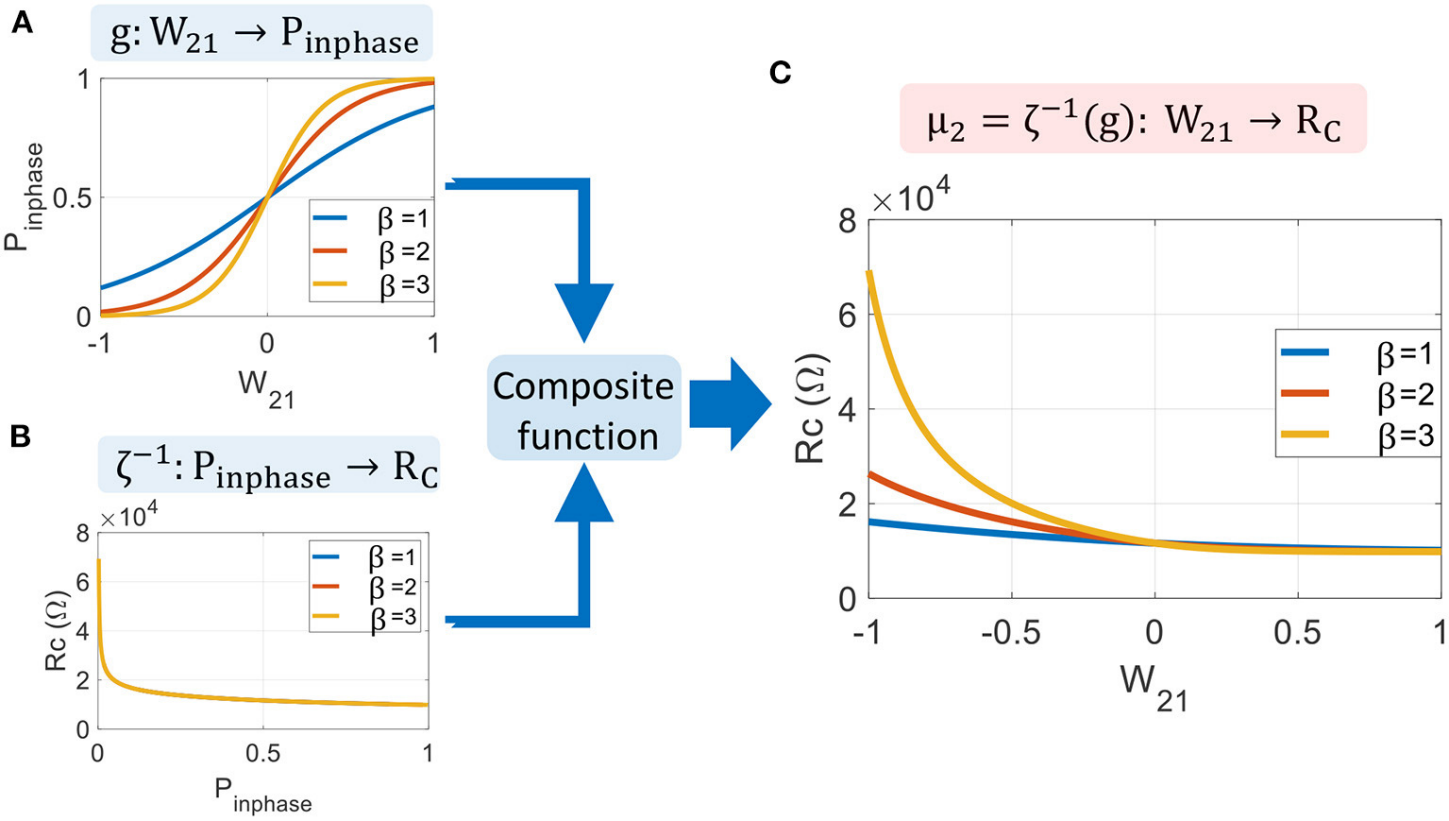
Mapping function for two coupled oscillators. **(A)** Sigmoid activation function presents the probability *P*_*inphase*_ for the two oscillators to be in phase. **(B)** Inverse of the transition function ζ^−1^ determines the coupling resistance *R*_*C*_ for a given probability *P*_*inphase*_. **(C)** The mapping function μ_2_ is obtained by the composite function ζ^−1^(*g*).

This mapping function is represented in Figure 11 for three different values of the sigmoid parameter, β. We see that this parameter sets the range of *R*_*C*_ and could be adapted for different ONN sizes. Interestingly, we notice that |Δ*W*_21_/Δ*R*_*C*_| is quite large for a positive weight, whereas it is much smaller for a negative one. For example, we see in Figure 11B that the function ζ^−1^ is a logarithmic function; thus, any small variation in Δ*R*_*C*_ around 10kΩ is likely to change the final phase state outcome. Whereas, for large *R*_*C*_, the two oscillators are almost always out-of-phase. This asymmetry in ζ^−1^ comes from the oscillator waveform type, as ζ^−1^ is derived from Equation (10), which is specific for relaxation oscillator waveform type. Hence, we expect some change for other types of waveforms, such as linear sawtooth, but the formulation of mapping (21) is general enough to be applied to any relaxation oscillators.

For large-scale ONN with N oscillators, we scale μ_2_ (21) by a factor *N*−1 to ensure the conservation of the current flow in coupling resistances. We finally obtain:


(22) $$\begin{array}{l} \mu_{N}\left(W_{ij}\right)=R_{ij}=\left(N-1\right)\zeta^{-1}\left(\frac{T_{osc}}{2}g\left(W_{ij}\right)\right) \end{array}$$

In next section, we demonstrate the effectiveness of the proposed mapping function (22) to design a 60-ONN architecture for pattern recognition as in HNN.

## 3. Results

### 3.1. ONN Design for Pattern Recognition

#### 3.1.1. ONN Training and Mapping

**Figure 12:**
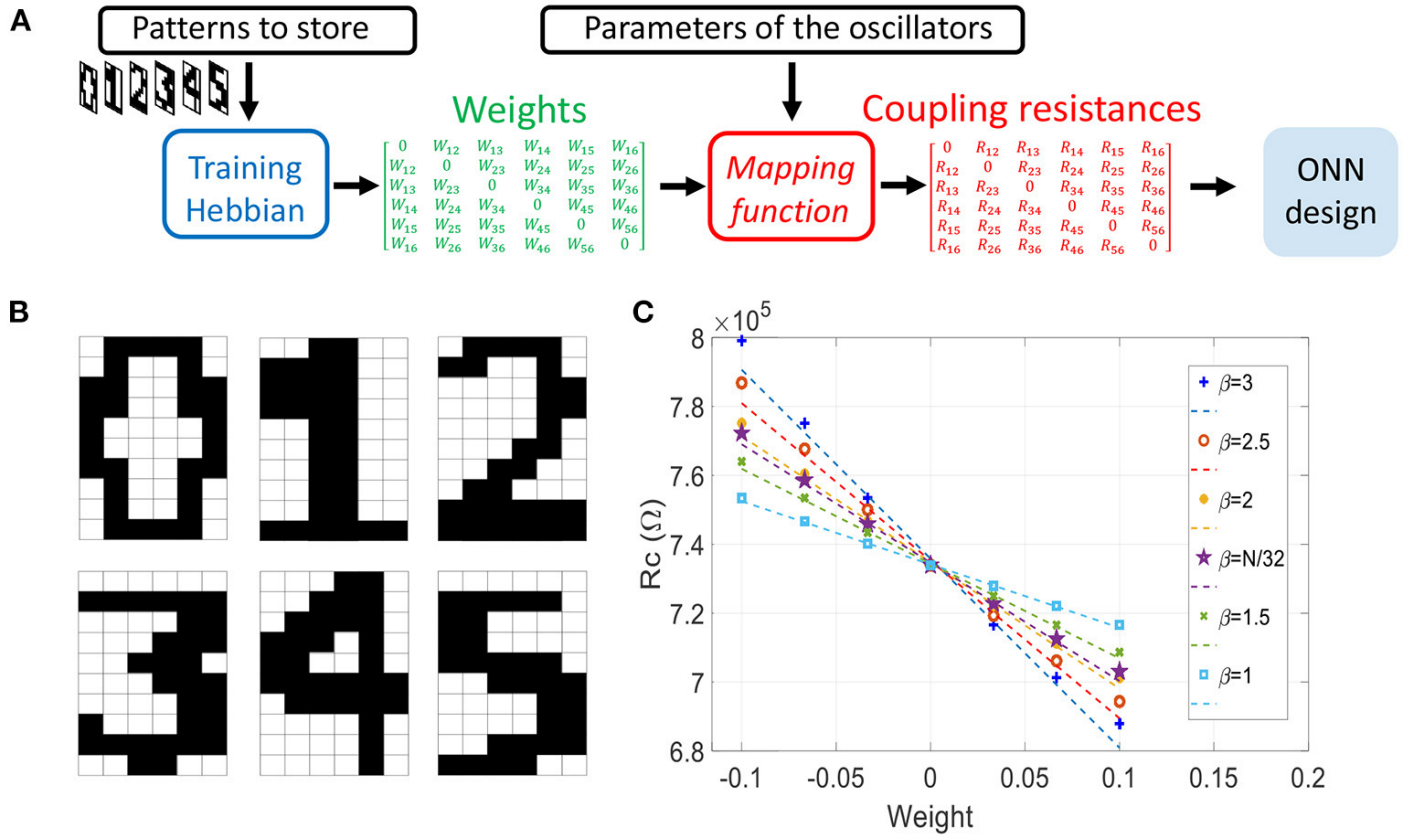
**(A)** Illustration of the ONN design flow for the associative memory application. Patterns to store can be represented as black and white images from which we compute weights with the Hebbian rule during the training process. Then, we use the mapping function μ_*N*_ to get the coupling resistances, allowing a systematic ONN design. **(B)** Stored patterns. **(C)** Coupling resistances as a function of Hebbian weights, computed with the mapping function μ_*N*_ for different values of parameter β.

In the previous section, we presented the memory capability of two coupled oscillators. Here, we apply the analytical formulas to a larger ONN size. We develop a design flow as shown in Figure 12A for pattern recognition with ONNs where we have implemented the proposed mapping function. We first compute the weights associated with the *M* stored patterns using the Hebbian Rule (Hoppensteadt and Izhikevich, 2000), as:


(23) $$\begin{array}{l} W_{ij}=\frac{1}{N}\overset{M}{\underset{k=1}{\sum }}\xi_{i}^{k}\xi_{j}^{k} \end{array}$$

We store *M* = 6 images representing digits “0”, “1” to “5” as shown in Figure 12B. Next, we use our mapping function to compute the coupling resistances associated with the Hebbian coefficients. The mapping is represented in Figure 12C for different values of parameter β which sets the slope of μ_*N*_(*W*_*ij*_). Increasing β induces a larger coupling resistance range. Because the Hebbian rule normalizes the weights by the network's size *N* (23), we scale β with *N* to keep a relative standard deviation of *R*_*ij*_ approximately constant when increasing the ONN size. By simulations, we found that the best accuracy is obtained for β = *N*/32, and we report the results in subsection 3.1.3.

#### 3.1.2. ONN Inference

**Figure 13:**
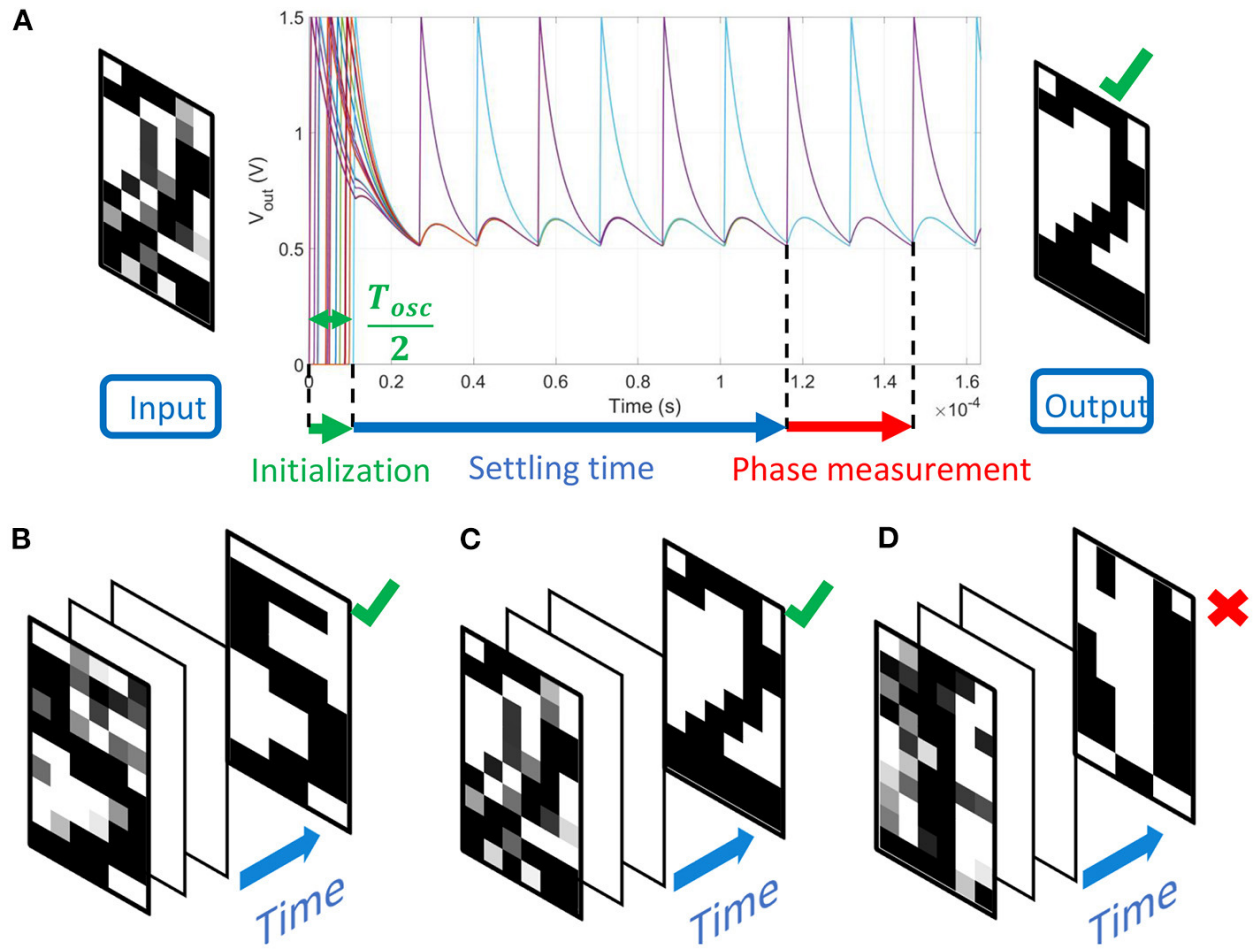
**(A)** Noisy input image “2” with 15 random altered pixels and voltage waveforms of 60 oscillators. ONN is initialized during *T*_*osc*_/2 with the noisy input image. After few oscillation cycles, ONN settles, and phases are measured. ONN retrieves the correct output image that corresponds to the stored pattern “2.” **(B)** The input image “5” has been altered at 15 random pixel locations by a uniform distribution. ONN retrieves the corresponding stored pattern. **(C)** Similarly, 15 random pixels of the input image “2” are altered, and ONN retrieves the correct corresponding pattern. **(D)** In this example, 20 noisy input pixels are introduced to digit “1,” and ONN converges toward a spurious state.

For every input, we set up the 60 ONN with black and white pixels encoded by –1 and +1, respectively. For pixels with black input, the corresponding oscillator is initialized with a delay Δ*t*_*init*_ = *T*_*osc*_/2 to set an initial out-of-phase relation (5) whereas, for a white pixel, no delay is introduced. A noisy gray pixel corresponds to an input delay between 0 and *T*_*osc*_/2. After few oscillations, the ONN settles, retrieves the noiseless pattern and phase relations are measured. An example of ONN voltage dynamics is presented in Figure 13A, showing the initialization and the settling time before the ONN stabilizes. Figures 13B,C show two examples of input images where 15 pixels have been randomly altered by a uniform distribution taking values between –1 and +1. When the number of noisy input pixels is too large such as in Figure 13D (20 noisy pixels), ONN converges toward a wrong spurious state that is different from the stored patterns. The results are in accordance with original observations from Hopfield (1982), proving that our mapping can implement HNN with ONN.

#### 3.1.3. ONN Recognition Accuracy

**Figure 14:**
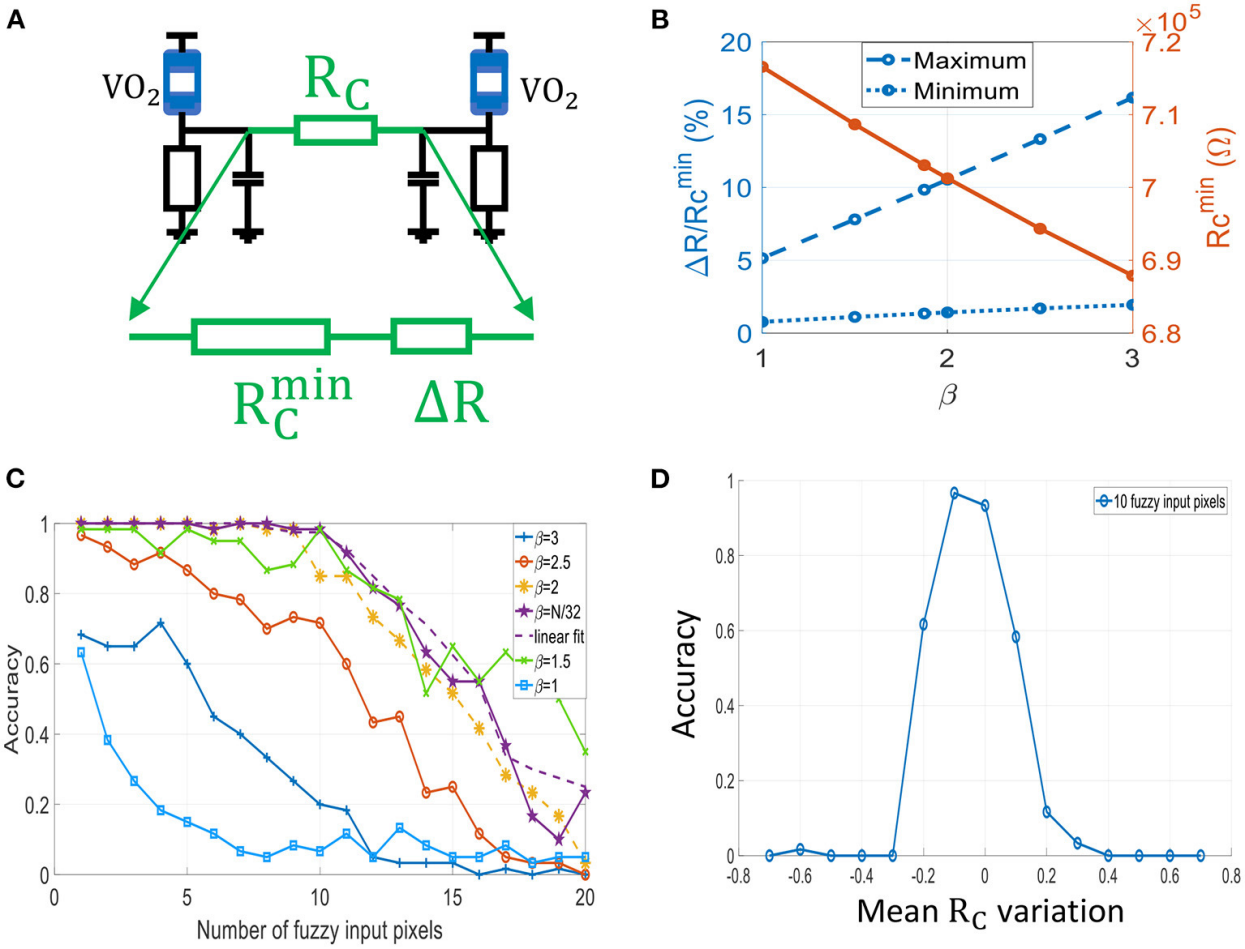
**(A)** Two oscillators coupled by *R*_*C*_, which can be decomposed in two series resistances: $R_{C}=R_{C}^{min}+\Delta R$. **(B)** Evolution of Δ*R*, $R_{C}^{min}$ with respect to β. $\Delta R^{max}\approx 10\%R_{C}^{min}$ gives the best accuracy results. Note that $\Delta R^{min}=1.7\%R_{C}^{min}$. **(C)** ONN recognition accuracy for different values of β. The dashed line is obtained for a linear fit of the mapping μ_*N*_, i.e., with coupling resistances that are linearly spaced. **(D)** Impact of *R*_*C*_'s mean variation on recognition accuracy.

Here, we perform simulations to compute the pattern recognition accuracy of the 60-ONN. We randomly apply noise to training patterns to generate a test set. It consists of 20 different subsets *S*_*k*_, *kϵ*{1, 2, .., 20} in which 60 different test patterns have *k* randomly located fuzzy input pixels. We vary the mapping function parameter β to assess its influence on ONN accuracy. We notice from Figure 14C that ONN achieves the best accuracy for an optimum value β = *N*/32 = 1.875. In this case, ONN recognizes more than 80% of test images with up to 20% of noise. As seen in Figure 12C, the slope parameter β sets the coupling resistance range, which in turn affects ONN accuracy. For instance, we observe that the set of coupling resistances obtained for β = 2 is similar to the case β = 2.5, but the accuracy is much lower in the latter case. In the next section, we quantify the coupling resistance accuracy that is required for synaptic design.

#### 3.1.4. ONN Coupling Resistance Range

We study the impact of *R*_*C*_'s relative variations and *R*_*C*_'s mean value. $R_{C}^{min}$ is the minimum resistance common to all coupling resistances, and Δ*R* is the additional series resistance to distinguish between weights (Figure 14A). Using the Hebbian rule, weights are located near “0” coefficient as in Figure 12C and our mapping function can be fitted linearly (dashed lines). Therefore, every coupling resistances can be approximated by $R_{C}\approx R_{C}^{min}+n\Delta R^{min}$ with *nϵ*{0, 1, 2, .., *M*}. Using this linear approximation, we can verify ONN accuracy is similar to the nominal case of mapping function with β = *N*/32, as shown in Figure 14C with the magenta dashed line.

As observed in previous sections, ONN accuracy is quite sensitive to the coupling resistances. We obtain $\Delta R^{max}\approx 15\%R_{C}^{min}$ for β = 3, and $\Delta R^{max}\approx 5\%R_{C}^{min}$ for β = 1 (Figure 14B). For these two cases as shown in Figure 14C, ONN shows poor accuracy. It is rather for β = *N*/32 = 1.875 with $\Delta R^{max}\approx 10\%R_{C}^{min}$ that ONN accuracy is above 90%.

To achieve the best ONN accuracy, a very good resistor matching is required, as we need a precision of $\Delta R^{min}=1.7\%R_{C}^{min}$ between two consecutive weights. To study the influence of the *R*_*C*_'s mean value only, we apply the same variation to all coupling resistances for β = *N*/32 and for 10 fuzzy input pixels (Figure 14D). We notice that the mean value of coupling resistances can vary from –10% up to +5% from its nominal value to achieve a similar accuracy.

## 4. Discussion

Oscillatory neural networks are triggering great interest for parallel processing applications, but a remaining challenge is how to compute with ONNs. To do so, we build analogies with ANN to determine the mapping between Hebbian learning coefficients (weights) to coupling resistors, knowing that they are essential elements for the network functionality. In this work, we proposed a mapping function that translates Hebbian signed weights to coupling resistances in a VO_2_-based ONN for systematic ONN analysis. Our simulations on 60-oscillators test case study highlighted a strong dependency between the ONN recognition accuracy and its coupling resistance range, set by mapping parameter β. Although we identified a suitable value β = *N*/32 to achieve good ONN accuracy, our mapping formulation provides coupling resistances that differ only with few percent. This would lead to significant hardware design constraints, as resistor mismatches smaller than 1.7% would be required to emulate two consecutive weights. In our mapping formalism, we used the phase transition function ζ, which provides the coupling resistance range holding the ONN memory. As we only derived ζ from oscillator dynamics, we believe the oscillator design could be optimized to expand the coupling resistance range and relax synaptic design constraints. For example, oscillator biasing current and supply voltage are the knobs that could be adjusted to maximize the synaptic range.

Here, we reported on a *mapping function* to compute coupling resistances from signed Hebbian coefficients in a VO_2_-based ONN. We first enhanced the ONN initialization control based on a simple architecture where every oscillator can be decoupled from the network via a switch. We were able to derive the phase *transition function* from the ONN dynamics, which is crucial for ONN memory. We then merged this analytical formulation with a sigmoid activation function from ANN formalism to build a mapping function. To demonstrate the ONN architecture's applicability with the proposed mapping function, we presented a test case of pattern recognition with 60 fully coupled oscillators. Finally, we showed that ONN recognition accuracy is very sensitive to relative variations between coupling resistances.

## Data Availability Statement

The raw data supporting the conclusions of this article will be made available by the authors, without undue reservation.

## Author Contributions

CD and AT-S developed the analytical formulation of ONN mapping and wrote the article. CD implemented the ONN circuit-solver framework on Matlab and performed simulations. Both authors contributed to the article and approved the submitted version.

## Funding

This work is supported by the European Union's Horizon 2020 research and innovation program, EU H2020 NEURONN (www.neuronn.eu) project under Grant No. 871501.

## Conflict of Interest

The authors declare that the research was conducted in the absence of any commercial or financial relationships that could be construed as a potential conflict of interest.

## Publisher's Note

All claims expressed in this article are solely those of the authors and do not necessarily represent those of their affiliated organizations, or those of the publisher, the editors and the reviewers. Any product that may be evaluated in this article, or claim that may be made by its manufacturer, is not guaranteed or endorsed by the publisher.
