# Supplementary material for: Mapping Hebbian Learning Rules to Coupling Resistances for Oscillatory Neural Networks
Source: Front Neurosci. 2021 Nov 8;15:694549. doi: 10.3389/fnins.2021.694549 (PMC8606813; doi:10.3389/fnins.2021.694549)
Supplement: Supplementary file 1 [file Presentation_1.pdf]

Supplementary Material

1 TRANSITION FUNCTION DERIVATION

1.1 Single oscillator time constants

Before studying the dynamics of two coupled oscillators to derive the transition function, we express the time constants of a single oscillator. The oscillating period T_{osc} is given by the sum of the charging and discharging time of the output capacitance:

$$T_{osc} = t_c + t_d \tag{S1}$$

If we assume abrupt VO_2 transitions we obtain:

$$t_c = \tau_c \log \left[\frac{V^- - V_{std}^{met}}{V^+ - V_{std}^{met}} \right] \tag{S2}$$

$$t_d = \tau_d \log \left[\frac{V^+ - V_{std}^{ins}}{V^- - V_{std}^{ins}} \right] \tag{S3}$$

With:

$$V_{std}^{met} = V_{DD} \frac{R_S}{R_S + R_{met}} \tag{S4}$$

$$V_{std}^{ins} = V_{DD} \frac{R_S}{R_S + R_{ins}} \tag{S5}$$

And with the two oscillator's time constants given by:

$$\tau_c = \frac{R_S R_{met}}{R_S + R_{met}} C_P \tag{S6}$$

$$\tau_d = \frac{R_S R_{ins}}{R_S + R_{ins}} C_P \tag{S7}$$

1.2 Dynamics of two coupled oscillators after initialization

The dynamics of two coupled oscillators can be expressed as:

$$\begin{cases} C_P \frac{dV_{out1}(t)}{dt} = \left(V_{DD1}(t) - V_{out1}(t)\right) G_{VO_2}^1(t) - \frac{V_{out1}(t)}{R_S} + I_{c1} \\ C_P \frac{dV_{out2}(t)}{dt} = \left(V_{DD2}(t) - V_{out2}(t)\right) G_{VO_2}^2(t) - \frac{V_{out2}(t)}{R_S} + I_{c2} \end{cases} \tag{S8}$$

To express the transition function $\zeta(R_C)$, we study the oscillators' dynamics until the first oscillator reaches the lower threshold V^- (before IMT). Then, the voltage difference between the two oscillators (set by the initial delay and R_C) determines the final phase state. We analytically solve (S8) for $t \geq t_{on}$ (when the two oscillators are coupled) until one output voltage reaches V^-. During our initialization procedure, we turn-on the two oscillators via V_{DD} and we couple them at time $t_{on} = \Delta t_{init} + t_c$. Therefore, both their output capacitors start to discharge. For this reason, at t_{on} the initial output voltages V^0 are given by

the dynamics of the uncoupled oscillators $i \in \{1,2\}$ when both VO_2 devices are in the insulating state: $R^i_{VO_2} = R_{ins}$:

$$V^0 = \begin{bmatrix} V^0_{out1} \\ V^0_{out2} \end{bmatrix} = \begin{bmatrix} V_{DD} \frac{R_S}{R_S+R_{ins}} \left[1 - \exp\left(-\frac{\Delta t_{init}}{\tau_d}\right) \right] + V^+ \exp\left(-\frac{\Delta t_{init}}{\tau_d}\right) \\ V^+ \end{bmatrix} \tag{S9}$$

It is convenient to rewrite (S8) in a matrix form to use the formalism from dynamical systems. As both oscillators are in insulating state, we can write the linear equation:

$$C \frac{dV}{dt} = G_A\, V + G_B\, V_{DD} \tag{S10}$$

With

$$V = \begin{bmatrix} V_{out1} \\ V_{out2} \end{bmatrix} \tag{S11}$$

$$G_A = \begin{bmatrix} -\frac{1}{R_{ins}} - \frac{1}{R_C} & \frac{1}{R_C} \\ \frac{1}{R_C} & -\frac{1}{R_{ins}} - \frac{1}{R_C} \end{bmatrix} \tag{S12}$$

$$G_B = \begin{bmatrix} \frac{1}{R_{ins}} & 0 \\ 0 & \frac{1}{R_{ins}} \end{bmatrix} \tag{S13}$$

$$C = \begin{bmatrix} C_P & 0 \\ 0 & C_P \end{bmatrix} \tag{S14}$$

$$V_{DD} = \begin{bmatrix} V_{DD1} \\ V_{DD2} \end{bmatrix} \tag{S15}$$

We solve the first-order differential equation (S10) given the initial conditions (S9). We consider t_{on} as the new time origin: $t - t_{on} \to t$ to express the solution as:

$$V = \left(\exp\left(C^{-1} G_A\, t\right) - I_d \right) G_A^{-1} G_B V_{DD} + \exp\left(C^{-1} G_A\, t\right) V^0 \tag{S16}$$

While both VO_2 devices are in insulating state, computing (S16) leads to:

$$\begin{cases} V_{out1} = \frac{V^0_{out1}}{2} \left(\exp(-\frac{t}{\tau_d}) + \exp(-\frac{t}{\tau'}) \right) + \frac{V^0_{out2}}{2} \left(\exp(-\frac{t}{\tau_d}) - \exp(-\frac{t}{\tau'}) \right) \\ \quad + V^{ins}_{std} \left(1 - \exp(-\frac{t}{\tau_d}) \right) \\ V_{out2} = \frac{V^0_{out1}}{2} \left(\exp(-\frac{t}{\tau_d}) - \exp(-\frac{t}{\tau'}) \right) + \frac{V^0_{out2}}{2} \left(\exp(-\frac{t}{\tau_d}) + \exp(-\frac{t}{\tau'}) \right) \\ \quad + V^{ins}_{std} \left(1 - \exp(-\frac{t}{\tau_d}) \right) \end{cases} \tag{S17}$$

The attraction between the two oscillators is characterized by the time constant:

$$\tau' = \frac{C}{\frac{1}{R_S} + \frac{1}{R_{ins}} + \frac{2}{R_C}} \tag{S18}$$

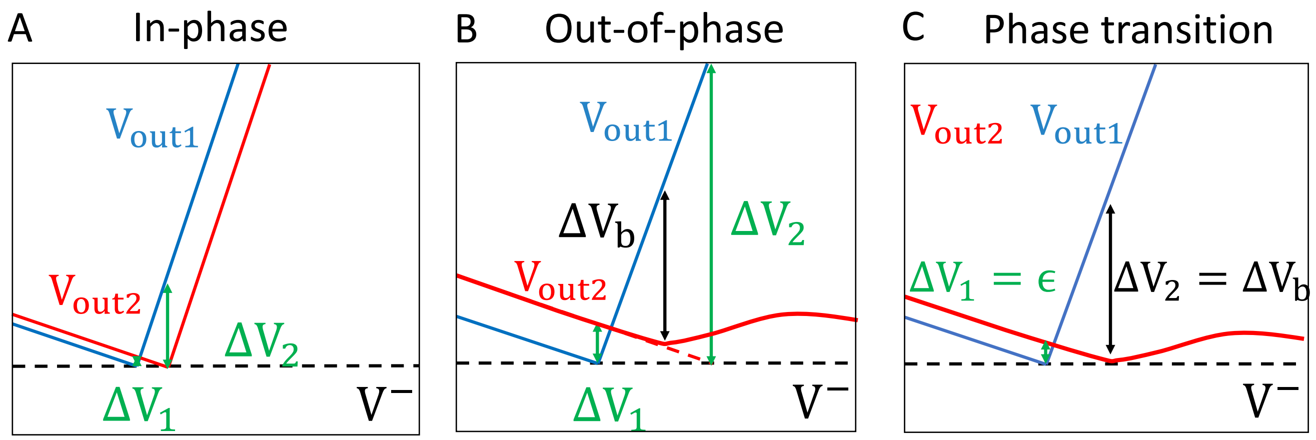

Figure S1. Output voltages near IMT threshold V^-. There are two final phase outcomes (A) in-phase state and (B) out-of-phase state. (C) The phase transition occurs when $\Delta V_2 = \Delta V_b$, and consequently when $\Delta V_1 = \epsilon$.

And we get a compact relation by expressing the difference:

$$\Delta V = V_{out2} - V_{out1} = \left(V^0_{out2} - V^0_{out1}\right) \exp\left(-\frac{t}{\tau'}\right) \tag{S19}$$

1.3 Final phase state and transition function

In simulations, when the first oscillator is about to reach V^- (Figure S1), we observe a voltage ϵ such that:

$$\begin{cases} \Delta V < \epsilon \rightarrow \Delta\phi_{out} = 0° \\ \Delta V \geq \epsilon \rightarrow \Delta\phi_{out} = 180° \end{cases} \tag{S20}$$

Using this observation, we derive the transition function ζ when both equations $\Delta V = \epsilon$ and $V_{out1} = V^-$ are fulfilled, as ϵ represents the transition voltage threshold between the two phase domains. To derive ϵ, we consider the metallic state of the oscillator, i.e. when C_P charges. Figure S1A and B illustrate the two scenarios that lead to in-phase or out-of-phase states. ΔV_1 and ΔV_2 are the voltage differences when V_{out1} and V_{out2} reach V^-, respectively. ΔV_b is the minimum voltage difference that pulls up V_{out2} (via synaptic current) and prevents it to reach the lower threshold V^-. This event is characterized by a "bump" appearing in the transient waveform, which postpones the IMT of oscillator 2. In this case, we have $dV_{out2}/dt = 0$, and using (S8), we obtain:

$$\Delta V_b = R_C\left(\frac{V^-}{R_{ins}} + \frac{V^-}{R_S} - \frac{V_{DD}}{R_{ins}}\right) \tag{S21}$$

This configuration, shown in Figure S1C, represents the limit case between A and B, and determines the phase transition. From this illustration, we express ϵ as:

$$\epsilon \approx \frac{s_1}{s_2}\Delta V_b \tag{S22}$$

with s_1, s_2 slopes of the discharge and charge curves, respectively. We approximate the slopes by considering the dynamic of a single oscillator, and we express them as:

$$\begin{cases} s_1 = \frac{V_{std}^{ins} - V^+}{\tau_d} \exp(\frac{-T_{osc}}{\tau_d}) \\ s_2 = \frac{V_{std}^{met} - V^+}{\tau_c} \end{cases} \tag{S23}$$

Using (S19), we express the time t_ϵ when $\Delta V = \epsilon$ as:

$$t_\epsilon = \tau' \log\left(\frac{V_{out2}^0 - V_{out1}^0}{\epsilon}\right) \tag{S24}$$

When the capacitor charge is much faster than its discharge ($s_2 >> s_1$), we obtain $\epsilon \to 0^+$. Hence, we can write $V_{out2}(t_\epsilon) = V_{out1}(t_\epsilon) + \epsilon \approx V_{out1}(t_\epsilon)$. The transition between the in-phase and out-of-phase domain occurs when:

$$V_{out1}(t_\epsilon) = V^- \to \frac{V_{out1}(t_\epsilon) + V_{out2}(t_\epsilon)}{2} \approx V^- \tag{S25}$$

By combining this last equation with (S17) and (S24), we finally express coupling resistances that describe the phase transition curve as:

$$R_C = 2\frac{R_S R_{ins}}{R_S + R_{ins}} \frac{\log\left(\frac{V^- - V_{std}^{ins}}{V_{out1}^0/2 + V_{out2}^0/2 - V_{std}^{ins}}\right)}{\log\left(\frac{\epsilon(R_C)}{V_{out2}^0 - V_{out1}^0}\right) - \log\left(\frac{V^- - V_{std}^{ins}}{V_{out1}^0/2 + V_{out2}^0/2 - V_{std}^{ins}}\right)} \tag{S26}$$
